# Use of Antigen Rapid Diagnostic Test for Detection of COVID-19 Cases in University Settings in Cameroon

**DOI:** 10.4269/ajtmh.23-0744

**Published:** 2024-09-03

**Authors:** Chavely Gwladys Monamele, Landry Messanga, Nicolas Njintang Yanou, Gustave Simo, Carole Else Eboumbou Moukoko, Henri Moumbeket, Abdou Fatawou Modyinyi, Ripa Mohamadou, Yacouba Foupouapouognigni, Armand Abdou, Jane-Francis Tatah Kihla Akoachere, Pallavi Dani, Anne Hoppe, Richard Njouom

**Affiliations:** ^1^ Department of Virology, Centre Pasteur Cameroon, Yaounde, Cameroon;; ^2^ Department of Biomedical Sciences, Faculty of Health Sciences, University of Buea, Buea, Cameroon;; ^3^ Department of Biological Sciences, Faculty of Science, University of Ngaoundere, Ngaoundere, Cameroon;; ^4^ Department of Biochesmistry, University of Dschang, Dschang, Cameroon;; ^5^ Department of Clinical Sciences, Faculty of Medicine and Pharmaceutical Sciences, University of Douala, Douala, Cameroon;; ^6^ Laboratory of Pharmacology and Toxicology, University of Yaounde I, Yaounde, Cameroon;; ^7^ Department of Agriculture, Livestock and By Products, National Advanced School of Engineering, University of Maroua, Maroua, Cameroon;; ^8^ Department of Microbiology and Parasitology, Faculty of Science, University of Buea, Buea, Cameroon;; ^9^ FIND, Geneva, Switzerland;; ^10^ Director of Innovation, Elizabeth Glaser Pediatric AIDS Foundation, Geneva, Switzerland

## Abstract

Robust testing strategies are an essential aspect of COVID-19 pandemic preparedness and response. In 2022, most regions of Cameroon were still below the WHO’s recommended level of 10 COVID-19 tests per 10,000 population. This study aimed to detect SARS-CoV-2 cases in university settings in Cameroon using antigen rapid diagnostic tests (Ag-RDTs) to increase national testing capacity and assess the knowledge, attitudes, and practices of this population regarding COVID-19 infection. Six universities in Buea, Douala, Dschang, Maroua, Ngaoundere, and Yaounde participated in this study from June to October 2022. Nasopharyngeal swabs were collected from participants and tested for COVID-19 using Ag-RDTs. For all positive cases, high-risk contacts were also tested by Ag-RDT. Participants were administered a structured questionnaire to assess their knowledge, attitudes, and practices regarding COVID-19. A total of 7,006 participants were recruited, and 54 (0.8%) were positive for SARS-CoV-2. Among close contacts, three of 62 (4.8%) tested positive. The University of Maroua was the only site to consistently report satisfactory testing capacity, achieving the study target of 30 tests/10,000 in 94.1% of cases. Participants’ knowledge of COVID-19 was moderate to good (≥50%). However, 28% were unsure about the effectiveness of the COVID-19 vaccine. Two main factors were identified that could facilitate the spread of SARS-CoV-2 in university settings, namely the lack of restrictions on entering campus without a mask (36%) and the non-respect of social distancing on campus (42.7%). The results of this study will guide future policies to better control diseases with epidemic or pandemic potential by targeting educational institutions.

## INTRODUCTION

The novel coronavirus disease 2019 (COVID-19), caused by severe acute respiratory syndrome coronavirus 2 (SARS-CoV-2), was first detected in Wuhan, China, in December 2019 and was declared a pandemic in March 2020.^1^ The virus spread worldwide, affecting different countries in different ways. Robust testing strategies were an essential aspect of preparedness and response to the COVID-19 pandemic, allowing for early detection of potentially infectious individuals and providing visibility on infection rates and transmission within communities.^2^ In addition, testing is a prerequisite for adequate contact tracing to limit spread through prompt identification. Reverse transcription real-time polymerase chain reaction, a nucleic acid amplification test (NAAT), remains the “gold standard” for COVID-19 diagnosis. However, antigen rapid diagnostic tests (Ag-RDTs), which detect the presence of viral proteins (antigens), have been used by countries to further strengthen their overall testing capacity, particularly in situations where NAAT capacity is limited.^3^ According to WHO standards, COVID-19 Ag-RDTs should have a minimum performance requirement of 80% sensitivity and 97% specificity.^3^

By June 2022, the third year of the COVID-19 pandemic in Cameroon, the incidence of COVID-19 had declined, with a positivity rate of 0.5%.^4^ The two recommended diagnostic methods were used in the population, and Ag-RDTs accounted for about 75% of the tests performed. The use of Ag-RDTs significantly increased testing by establishing mobile testing in market squares, schools, churches, and other public environments; however, most regions still did not reach the target of 10 tests/10,000 population as recommended by the WHO.^4^^,^^5^

At the time of this study in Cameroon, there was a relaxation of COVID-19 containment measures in the community, including the reopening of schools. However, independent measures were being taken by each educational institution to control the epidemic on its respective campus. These measures included a mix of face-to-face and virtual learning, temperature control on entry, physical barriers (masks), hand disinfection or handwashing with soap and water, and COVID-19 testing. Because schools bring individuals with very broad social connections to an environment where they share space and facilities, it was expected that transmission of SARS-CoV-2 would be higher in this population.^6^^,^^7^

This study aimed to assess the reliability of screening in the university setting as an efficient way to expand national testing capacity for SARS-CoV-2 and ensure a swift public health response. Because universities are expected to comprise a highly knowledgeable population, we also assessed the knowledge, attitudes, and practices of this population with regard to COVID-19 infection. This assessment was essential in order to understand the population and identify the gaps that can be addressed for better adoption of preventive measures.

## MATERIALS AND METHODS

### Study design.

This was a descriptive and cross-sectional study conducted from June to October 2022. Sociodemographic and clinical data were collected prior to specimen collection and COVID-19 screening performed with a SARS-CoV-2 Ag-RDT. Two different Ag-RDTs meeting WHO standards for satisfactory performance were used for screening: Biosynex (Biosynex Swiss SA, Freiburg, Switzerland) and Realy Tech (Hangzhou. China). Biosynex has a sensitivity of 96% and a specificity of 100% (https: www.biosynex.com), whereas Realy Tech has a sensitivity of 100% (clinical sensitivity of 95.4%) and a specificity of 97.9% (https://www.realytech.com). Individuals with positive results were considered positive for SARS-CoV-2, whereas asymptomatic individuals with negative results were considered negative. In accordance with national guidelines at the time the study was conducted, symptomatic individuals who tested negative for SARS-CoV-2 were either retested after 48 hours or referred for PCR testing. Close contacts of positive cases were traced and also tested by Ag-RDT. Close contacts were defined as all persons with whom the confirmed case shared indoor airspace or for whom there had been a prolonged period of face-to-face contact within the previous 5 days. All positive cases and contacts were referred to a designated treatment center for appropriate care in accordance with national guidelines.

We completed the testing component with an analytic and cross-sectional survey during the same period from June to October 2022 (Knowledge, Attitude, and Practice [KAP] study). Participants were administered a structured questionnaire to assess their knowledge, attitude, and practice regarding COVID-19. Participants were enrolled in the KAP study by consecutive sampling. The target population for the KAP study was similar to that for the COVID-19 screening study and consisted of students, staff, and people who frequently visited the university campus. All individuals who were willing to participate were enrolled.

### Study population and setting.

Cameroon is a Central African country with a population of approximately 27 million inhabitants. More than half of the population is between 15 and 65 years of age, with a male-to-female sex ratio of 1.0009.^8^ There are eight public universities and several private higher education institutions spread throughout the country.

The study population included all students, staff (academic and nonacademic), or other persons who frequently visited the university campus. Close contacts of positive cases were screened, whether they were on campus or off campus. Participants enrolled in the KAP assessment were not required to participate COVID-19 screening study and vice versa.

Six universities in Cameroon (five state universities and one private institution) participated in this study: the Universities of Buea, Douala, Dschang, Maroua, and Ngaoundere and the Institut Universitaire Protestant de Yaoundé (IUPY) (Table 1). To scale up testing capacity at the national level, we set a weekly target of an average of 30 tests/10,000 people in each of the universities.^9^^–^^14^

**Table 1:** Cameroon university population

University	Population	Minimum Number of Tests per Week	References
University of Buea	12,000	36	University of Buea^9^
University of Douala	40,000	120	University of Douala^10^
University of Dschang	14,000	42	Universite de Dschang^11^
University of Maroua	12,000	36	University of Maroua^12^
University of Ngaoundere	30,000	90	University of Ngaoundere^13^
IUPY	12,000	36	–
Total	120,000	360	–

IUPY = Institut Universitaire Protestant de Yaoundé.

### Data collection, management, and analysis.

In each university, teams of data collectors were positioned in different areas . Flyers were posted on university notice boards to publicize the testing campaign. Data from participants were entered directly into a structured questionnaire on an online platform (Kobo Toolbox; https://www.kobotoolbox.org/) using a smartphone. The questionnaire used for screening included information on participant identification; sociodemographics, clinical and travel histories, and vaccination status, whereas the questionnaire used for the KAP analysis consisted of four components: respondents’ demographic data; knowledge of COVID-19, including risk factors, transmission, and prevention; attitudes toward COVID-19 in terms of perceived susceptibility, beliefs, and risks of having COVID-19; and the practice of preventive measures or the presence of communal measures to limit the spread of COVID-19, such as vaccination, social distancing, and presence of handwashing stations on campus. The classification of responses as “low,” “moderate,” or “good” was based on the percentage of correct responses: below 50%, 50–69%, and above 70%, respectively. All data collected were coded to ensure confidentiality. The Kobo Toolbox used for data collection interfaced with real-time analysis of submitted data, ensuring the completeness of all data entered. Statistical analyses were carried out using SPSS software (IBM, Chicago, IL). *P*-values below 0.05 were used to determine the level of significance between the presence of COVID-19 infection and participant characteristics.

### Stakeholder engagement activities.

Prior to the start of the project, engagement was carried out with some departments of the Ministry of Public Health to inform them of the study objectives and how the results would be disseminated. In particular, engagement was carried out with the Department for the Control of Diseases, Epidemics and Pandemics (DLMEP) and the Center for Coordination of Public Health Emergency Operations (CCOUSP).

## RESULTS

### Participants’ characteristics.

**Figure 1.**
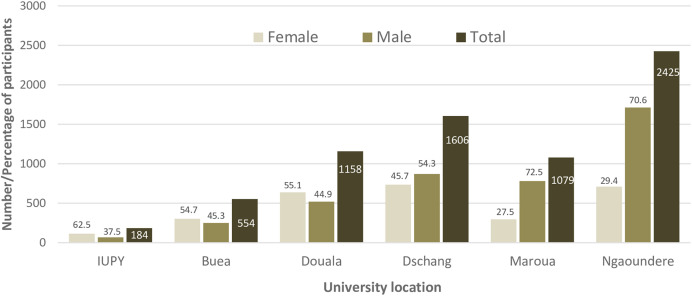
Sex distribution of participants in the universities. In terms of faculty, participants from the Faculty of Science were the most represented at 29.2%, followed by Faculty of Engineering and Technology (16.6%), then by the Faculty of Arts, Letters, and Humanities (Figure 2). IUPY = Institut Universitaire Protestant de Yaoundé.

A total of 7,006 participants were recruited from the six universities participating in this study irrespective of the presence of respiratory symptoms suggestive of COVID-19. There were more males (60%) than females (40%), with a male-to-female ratio of 1.5 (Figure 1). The mean age of the participants was 22.4 years. Students were the most represented population at 96.2%, followed by support staff (0.8%) and teachers (0.8%). The majority of the participants were asymptomatic (93.0%), and 92.5% had never been vaccinated against SARS-CoV-2.

**Figure 2.**
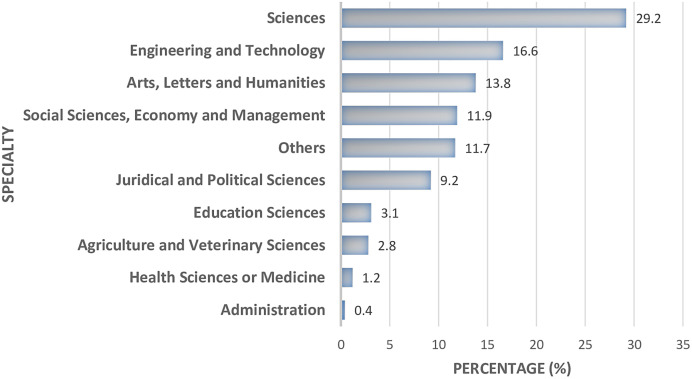
Distribution of participants by faculty.

### Testing capacity in the universities.

The number of tests administered at each university ranged from 184 at IUPY to 2,425 at the University of Ngaoundere (Table 2). The universities of Dschang, Maroua, and Ngaoundere were the three sites that achieved the overall expected number of tests, although this was not evenly distributed across all study weeks. The average number of tests/10,000 in the university setting was 53 (range: 30–77), and the weekly target of 30 tests/10,000 population was achieved 79.7% of the time across all universities in the sample. Meanwhile, the WHO-recommended target of 10 tests/10,000 population was achieved in 92.2% of weeks. The University of Maroua was the only site to consistently report satisfactory testing capacity. This university achieved the study target of 30 tests/10,000 population in 94.1% of weeks and the WHO-recommended target of 10 tests/10,000 population in all weeks.

**Table 2:** Tests performed and testing capacity in the university settings

University Location	Weekly Target	Overall Expected	Total Tests Performed	Average Weekly Tests/10,000	Weeks with 30 Tests/10,000 *N* (%)	Weeks with 10 Tests/10,000 *N* (%)
Buea	36	576	554	38	7 (58.3)	11 (91.7)
Douala	120	1,920	1,158	30	5 (55.6)	6 (66.7)
Dschang	42	672	1,606	72	9 (56.3)	14 (87.5)
Maroua	36	576	1,079	53	16 (94.1)	17 (100)
Ngaoundere	90	1,440	2,425	48	12 (70.6)	15 (88.2)
IUPY	36	576	184	77	02 (100)	02 (100)
Total	360	5,760	7,006	53	51 (79.7)	59 (92.2)

IUPY = Institut Universitaire Protestant de Yaoundé.

### Positivity rate among university participants and among close contacts of positive cases.

Of the 7,006 participants recruited, 54 (0.8%) were found to be positive for SARS-CoV-2. The University of Ngaoundere had the highest positivity rate (1.2%), whereas the University of Maroua had no cases of SARS-CoV-2 (Table 3). Sixty-two close contacts of the positive cases were tested for SARS-CoV-2, and three were positive (4.8%). Of these, 15 (24.2%) were contacts outside the university environment. Two of the positive contacts were school contacts, whereas the other was a healthcare worker (nurse).

**Table 3:** SARS-CoV-2 positivity rate in university settings and in primary contacts

University	University Participants		Primary Contacts of Positive Cases	
	Tested	Positive *N* (%)	Tested	Positive *N* (%)
University of Buea	554	4 (0.7)	10	0
University of Douala	1,158	13 (1.1)	3	0
University of Dschang	1,606	9 (0.6)	38	3 (7.9)
University of Maroua	1,079	0	0	0
University of Ngaoundere	2,425	28 (1.2)	11	0
IUPY	184	0	0	0
Total	7,006	54 (0.8)	62	3 (4.8)

IUPY = Institut Universitaire Protestant de Yaoundé.

Table 4 shows the association between the presence of SARS-CoV-2 and some participants’ characteristics. Significantly higher COVID-19 positivity rates were observed in symptomatic individuals (4.5%) and in the month of July (1.7%), whereas a higher COVID-19 positivity rate was also detected in support staff (3.4%); no conclusions could be drawn from two individuals.

**Table 4:** Association between COVID-19 and sociodemographic data

Characteristics	Negative *N* (%)	Positive *N* (%)	*P*-Value
Mean Age	22.4 ± 5.4	23.7 ± 7.1	–
Age Group (years)			
<21	3,540 (99.4)	23 (0.6)	0.244
21–40	3,284 (99.2)	28 (0.8)	
>40	128 (97.7)	3 (2.3)	
Sex			
Male	4,168 (99.1)	38 (0.9)	0.252
Female	2,784 (99.4)	16 (0.6)	
Position			
Student	6,693 (99.3)	48 (0.7)	0.033*
Teacher	58 (98.3)	1 (1.7)	
Support Staff	56 (96.6)	2 (3.4)	
Others	145 (98.0)	3 (2.0)	
Status			
Symptomatic	469 (95.5)	22 (4.5)	<0.001*
Asymptomatic	6,483 (99.5)	32 (0.5)	
Travel History			
Yes	947 (98.7)	12 (1.3)	0.186
No	6,005 (99.3)	42 (0.7)	
Vaccination Status			
Yes	203 (98.5)	3 (1.5)	0.288
Incomplete	318 (99.7)	1 (0.3)	
No	6,431 (99.2)	50 (0.8)	
Past Infection with COVID-19			
Yes	27 (96.4)	1 (3.6)	0.232
No	6,925 (99.2)	53 (0.8)	
Month			
June	1,292 (99.8)	2 (0.2)	<0.001*
July	1,047 (98.3)	18 (1.7)	
September	1,659 (99.8)	4 (0.2)	
October	2,954 (99.0)	30 (1.0)	
Period			
End of Academic Year	2,339 (99.2)	20 (0.8)	0.041*
Beginning of Academic Year	4,613 (99.3)	34 (0.7)	

* Statistically significant difference.

### Assessment of KAP regarding SARS-CoV-2 in universities.

A KAP questionnaire was used to collect information from 3,989 participants to assess their knowledge, attitude, and practice with respect to COVID-19 in the university setting. The mean age of the participants who took part in this survey was 23.3 years (range: 14–91 years), with a higher proportion of males (66.2%).

### Knowledge of COVID-19.

Participants’ knowledge of COVID-19 was moderate to good (≥50%). Participants had good knowledge of the modes of transmission of COVID-19: 74.8% knew that the COVID-19 virus is spread by respiratory droplets from infected people when sneezing, coughing, or talking, whereas 75.2% knew that the COVID-19 virus is spread by direct contact with contaminated hands, fomites, and surfaces. In all, 52.6% believed that vaccination protects against COVID-19, whereas 28% were unsure about the effectiveness of the vaccine (Table 5).

**Table 5:** Assessment of knowledge on COVID-19 in university settings

Knowledge of COVID-19	False	True	Don’t Know
Transmission of COVID-19 is not possible when signs and symptoms are not present	**55.2**	18.6	26.1
It is not necessary for young adults to take measures to prevent COVID-19	**69.0**	13.9	17.0
COVID-19 virus spreads via respiratory droplets of infected individuals during sneezing, coughing, or speaking	8.3	**74.8**	16.9
COVID-19 virus spreads via direct contact with contaminated hands, fomite, and surfaces	9.2	**75.2**	15.6
Vaccine protects against COVID-19	19.4	**52.6**	28.0

The most represented responses are shown in boldface font.

**Figure 3.**
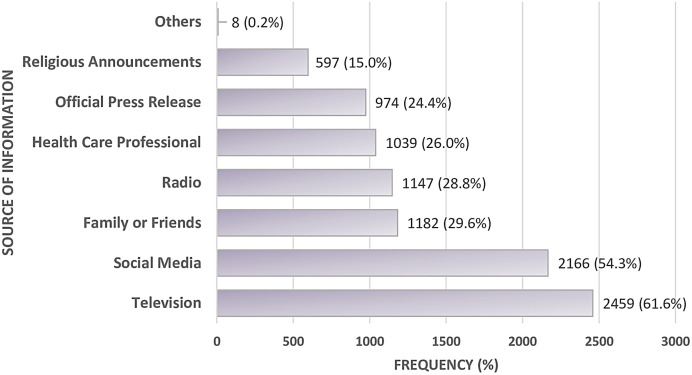
Source of information about SARS-CoV-2.

Regarding the source of information about SARS-CoV-2, most participants received information from television and social media. Smaller proportions received information from the radio, family/friends, health professionals, and official press releases (Figure 3).

### Attitudes toward COVID-19.

Participants’ perception of COVID-19 was low to moderate. Most participants thought that COVID-19 was a serious disease (59.1%), whereas 30.4% of participants admitted that they did not care about the disease and were continuing their normal activities. Although not unanimously accepted by all participants, the majority believed that government measures, including recommended handwashing, social distancing, avoidance of crowded places, wearing a face mask, avoidance of handshaking, and quarantine were effective in reducing the spread of COVID-19. One-third of participants believed that COVID-19 testing was recommended only in the presence of symptoms (Table 6).

**Table 6:** Assessment of attitude on COVID-19 in university settings

Perceived Susceptibility and Beliefs with Respect to COVID-19	No	Rarely	Often	Yes
I think COVID-19 is a serious disease if it is not prevented	11.4	8.7	20.9	**59.1**
I believe that recommended handwashing is helpful for me, to protect myself from COVID-19	8.8	6.9	14.4	**69.9**
I believe that recommended social distancing is helpful for me, to protect myself from COVID-19	9.4	7.0	16.2	**67.4**
I believe that avoidance of crowded places is helpful for me to protect myself from COVID-19	13.8	8.4	18.2	**59.6**
I believe that wearing face masks is helpful for me, to protect myself from COVID-19	8.9	6.6	17.1	**67.3**
I believe that not shaking people’s hands is helpful for me, to protect myself from COVID-19	12.3	9.3	19.7	**58.8**
If I or one of my family members or a friend is infected with COVID-19, self-isolation is needed	13.0	6.8	14.9	**65.3**
I need a COVID-19 test only if I have symptoms	**48.6**	7.2	11.0	33.2
I think that I am susceptible to COVID-19	**46.9**	13.8	12.3	27.0
I don’t care about this disease and do my daily activities like before	**36.6**	10.5	22.6	30.4

The most represented responses are highlighted in boldface font.

### Assessment of individual practices and societal measures to limit spread of SARS-CoV-2.

**Figure 4.**
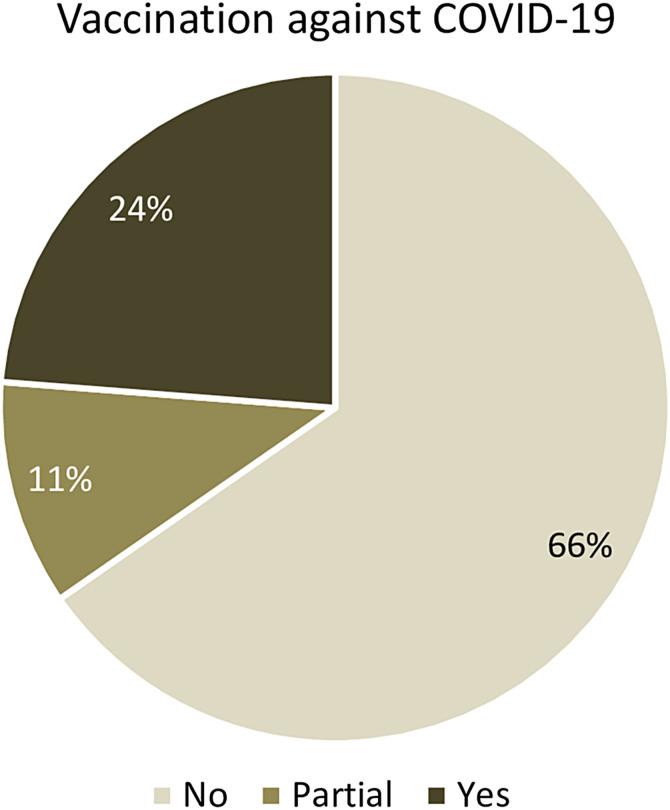
COVID-19 vaccine uptake in the university study sites in Cameroon.

Based on participants’ responses, the majority practiced measures that could limit the spread of SARS-CoV-2, including covering the mouth and nose when coughing or sneezing (52.6%), practicing recommended handwashing (46.1%), and using hand sanitizers (39.8%) (Table 7). Few participants practiced physical distancing (17.6%) or had received a vaccination against COVID-19 (23.5%) (Figure 4).

**Table 7:** Factors favoring transmission of SARS-CoV-2 in university settings

Practice of Individual Factors	No	Rarely	Often	Yes
Covering Mouth and Nose When Coughing or Sneezing	7.7	11.8	27.9	**52.6**
Practicing Recommended Handwashing	10.0	14.3	29.6	**46.1**
Use of Hand Disinfectants	13.1	16.6	30.6	**39.8**
Avoiding Touching Eyes, Nose, and Mouth with Unwashed Hands	14.7	20.2	35.0	**30.1**
Wearing of a Face Mask When Going to Crowded Areas	16.5	20.6	**36.4**	26.5
Practicing Physical Distancing at Least 1 m Away from Others	21.5	24.8	**36.2**	17.6
Practicing Disinfecting Surfaces Belonging to Me (e.g., phone)	21.4	23.5	**33.1**	22.0
Staying at Home When Sick or When Having a Cold	19.9	21.2	**31.7**	27.2
Factors Specific to University Setting				
Availability of Masks around Campus (to buy)	19.4	14.7	24.1	**41.9**
Presence of Handwashing Stations on Campus	25.7	17.2	17.2	**40.0**
Presence of Hand Sanitizers on Campus	27.3	17.8	26.0	**28.9**
Respect of Social Distancing in Class	**42.7**	19.7	21.7	15.8
Restrictions on Campus If Absence of Mask	**36.0**	19.5	24.6	19.8
Presence of Posters on Campus for Information on SARS-CoV-2	**32.3**	16.0	19.4	32.2

Bold face font indicate the most represented responses.

Some measures to limit the spread of SARS-CoV-2 were already in place in the university setting. These included the availability of masks on campus (for sale) and the presence of handwashing stations and hand sanitizers on campus. In contrast, factors that could facilitate the spread of SARS-CoV-2 were also identified. These included the lack of social distancing in the amphitheaters (42.7%), the lack of restrictions on entering the campus without a mask (36%), and the lack of posters on campus with information about SARS-CoV-2 (32.3%).

## DISCUSSION

In this study, 7,006 participants were recruited from six universities with a sex ratio slightly in favor of males, compared with the overall sex ratio of 1.13 in universities in Cameroon^15^ and 1.00 in the general population.^8^ Participants from the Faculty of Science and the Faculty of Engineering and Technology were more likely to be tested for SARS-CoV-2, whereas participants from the Faculty of Medicine/Health Sciences were less likely to be tested, suggesting the fear of medical personnel to test for SARS-CoV-2.^16^

In the six universities in Cameroon that participated in this study, an overall positivity rate of 0.8% was observed. This low rate is consistent with global statistics,^17^ as a majority of countries experienced a decreasing trend in the number of new COVID-19 cases, particularly in Cameroon.^4^ Three cases were detected among the close contacts of the confirmed cases. Two of these positive contacts were from the same university, and one was a healthcare worker. These results confirm the hypotheses that university settings provide an environment for efficient transmission of COVID-19 and that barrier measures should be respected to limit transmission of COVID-19 or other respiratory pathogens of public health importance. A major limitation to an effective public health response was the fact that tracing and screening of high-risk contacts was not performed for all positive cases owing to the reluctance of cases to list all their contacts. Also, the fact that the project did not plan for the treatment or management of positive cases within the universities resulted in the loss to follow-up of cases. Although the overall positivity rate in this study was low (0.8%), significantly higher COVID-19 positivity rates were recorded in symptomatic individuals (4.5%), and in the month of July (1.7%). This may be explained by the increase in cases due to the BA.4 and BA.5 omicron variants, which caused the fifth wave of the pandemic in Cameroon in the month of August 2022.^18^

Testing in university settings using Ag-RDT did not achieve the targeted testing capacity. Only the University of Maroua achieved the study target of 30 tests/10,000 in 16/17 (94.1%) weeks and the WHO-recommended testing capacity of 10 tests per 10,000 in all weeks. Although most sites did not meet the required testing target, strategies to ensure continuous testing in all tertiary institutions will significantly contribute to increasing the country’s testing capacity for rapid detection of cases and allow a swift response to localized outbreaks. This was also confirmed in a previous study conducted in a university setting in California.^19^ In asymptomatic testing scenarios, increased testing frequency and rapid turnaround time should be prioritized over test sensitivity.

Owing to various sources of information, most commonly television and social media, the university population had satisfactory knowledge of COVID-19, especially about the modes of transmission, similar to other studies conducted in sub-Saharan Africa.^20^ However, knowledge about the efficacy of the COVID-19 vaccine was moderate, with only about half of participants convinced of its efficacy. This is probably due to concerns about the perceived safety of the COVID-19 vaccines and the lack of evidence-based information on the vaccines as reported in previous studies.^21^^,^^22^ However, these vaccines have been shown to be effective in reducing rates of infection, disease severity, hospitalization, and mortality in the population.^23^ Reports of COVID-19 vaccination coverage indicate that approximately 5–10% of the target population in Cameroon had been vaccinated as of December 2022, well below the 70% target.^24^^,^^25^ In our study, up to 23.5% of the participants were vaccinated against COVID-19, which is two to four times higher than the national average, although still lower than expected. The relatively high proportion in our study population could be explained by the higher level of education of the study participants, who were therefore more aware of the importance of vaccination. However, there is still a need to intensify vaccination campaigns and communication strategies that can easily reach the community.

One-third of the study participants thought that the COVID-19 test was only necessary if symptoms were present. This is reflected in the overall moderate testing rates in university settings. Participants were very reluctant to agree to participate. However, the distribution of masks and disinfectant gel was a strong motivator to increase testing. In contrast, in a study conducted in a high-income setting, internal motivation and convenience were prominent factors supporting testing uptake, whereas gift incentives increased community testing, particularly among people who had never been tested.^26^ Awareness raising on SARS-CoV-2 should be conducted in university and other educational settings to emphasize the importance of regular testing of asymptomatic individuals, as they are potential carriers of the SARS-CoV-2 virus.

With regard to the practice of preventive and barrier measures, only a few participants or institutions still practiced social distancing. In the same vein, some institutions have ceased restricting the mandatory wearing of face masks for all those entering the university campus. These findings are consistent with a previous review that found limited practice of preventive measures in low-income countries.^27^ A study conducted in Ethiopia found that the main barriers to effective implementation of public health measures were resistance to change, negligence, lack of community involvement, insufficient training of frontline workers, poor supportive supervision, poor law enforcement, and lack of continuous community awareness.^28^ In addition, a study performed in Cameroon reported that scientific, social, and cultural factors were associated with nonadherence to preventive measures. These factors included the belief that the COVID-19 disease is not as serious in Africa, the belief that it is a political disease, the belief that protection can be achieved through the use of natural or traditional medicines, and difficulty in respecting social distancing by some cultural groups or professions (Muslims, healthcare workers, and traders).^29^

## CONCLUSION

In conclusion, testing in university settings using Ag-RDT did not prove sufficient to achieve study testing capacity, as only one university achieved satisfactory testing performance. The positivity rate in the university setting and among the contacts of the positive cases was low, similar to the decreasing trend observed worldwide. Information about the COVID-19 vaccine was a source of ambiguity, with most respondents doubting its efficacy. Failure to implement some individual or communal measures, including not restricting people from entering the campus without a mask and not observing social distancing on campus, are risk factors that could lead to an increase in COVID-19 cases in university settings. Awareness and advocacy campaigns about SARS-CoV-2 would increase knowledge about the disease and minimize the risk of infection in university and other educational settings. In May 2023, the WHO declared that COVID-19’s status as a public health emergency had ended.^30^ The results of this study will therefore guide future policies to better control diseases with epidemic or pandemic potential by targeting specific groups such as educational institutions.
